# Research on the ordering strategy problem in supply chain with profit allocation under two-level price-fluctuation sales

**DOI:** 10.1371/journal.pone.0316377

**Published:** 2025-01-14

**Authors:** Minchao Zheng, Ying Yu, Jiayang Xu, Yunzhi Mu

**Affiliations:** 1 School of Economics and Management, Zhejiang University of Science and Technology, Hangzhou, Zhejiang, China; 2 College of Economics and Management, Zhejiang Normal University, Jinhua, Zhejiang, China; 3 School of Business Management, Zhejiang Financial College, Hangzhou, Zhejiang, China; University of Madeira / NOVA Lincs, PORTUGAL

## Abstract

This paper investigates optimal ordering strategies in supply chains under two-level price fluctuations and initial profit allocation. By utilizing Copula functions to model the complex relationship between fluctuating prices and uncertain demand, the study develops both continuous and discrete decision models for practical applications. A discrete algorithm is proposed to approximate the optimal solution, with its convergence rigorously proven. Numerical experiments demonstrate that profit allocation ratios significantly impact optimal order quantities and overall supply chain profit. Price fluctuations, particularly at the discount level, present critical challenges, necessitating flexible and adaptive ordering strategies. The study also investigates the influence of different Copula relationships on optimal ordering decisions, revealing how varying market conditions—from moderate price sensitivity to high volatility—affect optimal order quantities. By examining ordering strategies in the context of profit allocation contracts, this research offers a new perspective on how supply chain members can collaboratively navigate uncertain markets. The findings provide actionable insights for managers to mitigate risks, improve coordination, and seize new opportunities. Extending traditional models to incorporate price fluctuations and profit allocation, this study makes theoretical and practical contributions to supply chain management, offering robust strategies to strengthen supply chain resilience.

## 1. Introduction

As markets evolve rapidly, an increasing number of products now encounter shorter life cycles, with the resulting surplus intensifying competition, which prompts retailers to adjust prices frequently to sell products quickly and capture more market share [1–3]. In China, for instance, clustered commercial and agricultural trade markets are typical examples of markets where products have short life cycles and fierce retail price fluctuations influenced by purchase volume, bargaining powers, competition from similar products, and supply. Similarly, online sales also undergo price fluctuations driven by advanced technology and have to adjust real-time prices as frequently as possible [4, 5]. Therefore, retail prices vary across customers and time, an uncertain random variable known as random price fluctuation. In addition, retailers often implement significant price reductions in the sales cycle based on sales timing and inventory levels, leading to price fluctuations within a discount range to attract customers and boost revenues [6]. This strategy is common among fast-moving consumer goods like Only, Semir, and Metersbonwe, where products are sold at their original prices and significantly reduced prices in later stages of sales to clear stock and maximize profits as they are out of season, or inventory accumulates [7, 8]. Staged price fluctuations and demand uncertainty are common, presenting significant challenges for retailers’ ordering decisions [9, 10]. Given these complexities, it is essential to explore effective ordering strategies that navigate such price fluctuations.

The research on ordering problems considering retail prices can be roughly divided into two categories: one focusing on price adjustment strategies and the other examining passive price fluctuations. The former sets a marketing model that ties retail prices with certain sales strategies, where the same product is sold at different retail prices either at the same sales stage or across stages. Some studies discuss the ordering problem based on pre-determined fixed price strategies, such as multi-price policies [11–13], membership pricing [14], retail prices across channels [15], gift cards [16], and quantity discounts [17]. Strategies such as customer segmentation, product bundling, clearance pricing, and purchasing scenarios are also considered [18–20]. Some others focus on dynamic price adjustments, where retailers adjust prices and determine ordering strategies based on actual sales, and this has gained significant attention in recent years [21]. For example, Bora Keskin et al. [22] studied how dynamic pricing helps retailers of perishable products manage inventory pressures; Li and Shinji [23] used online learning algorithms to approximate optimal solutions under dynamic pricing strategies; Herbon et al. [24] proposed an optimal replenishment plan and dynamic pricing model where prices influence demand in an additive manner; Pang et al. [25] discussed a periodic review inventory strategy under dynamic price adjustments. The other price-related research deals with passive price fluctuation, primarily used in industries where external markets heavily influence prices [26, 27]. With this approach, companies determine appropriate ordering strategies by predicting price fluctuations to mitigate risks. Although relatively few studies have focused on this area, it has gained some attention in recent years, with existing research typically addressing ordering problems by predicting price distributions and the joint probability distribution with price and demand. For instance, Tapiero et al. [28] analyzed a competitive market scenario where retailers set predicted price distributions without knowing actual market price changes, examining the interplay between risk aversion, subjective retailer evaluations, and market-implied risk-neutral strategies. Zheng et al. [29] considered the uncertainty of both price and demand within the Stackelberg competition framework.

As a member of the supply chain, retailers’ ordering decisions are influenced not only by market fluctuations but also by stakeholders within the supply chain [30–32]. Haitao Cui et al. [33] observed that supply chain members refer to each other’s profits. When they perceive an unfair profit allocation, they may even undermine their interests to impact others, potentially disrupting cooperation [34]. Therefore, reasonable profit allocation is crucial in the decision-making processes of supply chain members.

Two forms of profit allocation contracts are commonly used in supply chains. One is the profit-sharing contract, where suppliers provide products to retailers at low wholesale prices, and then retailers return profits to suppliers based on a decentralized decision-making framework [35–38]. This type of contract has been extensively studied in recent years. Uncertain market conditions, including demand uncertainty, price changes, and changing policies, are considered. For example, Dehghan-Bonari et al. [39] investigated a green supply chain where revenue-sharing contracts were adopted under stochastic independent demand to encourage a retailer to increase its order quantity. Yao et al. [40] also studied the impact of price-dependent stochastic demand on cooperative effects in supply chains using revenue-sharing contracts under the classic newsvendor model and found that revenue-sharing contracts perform better than price-only contracts and the benefits earned by different supply chain partners vary due to demand variability and price sensitivity. Different pricing strategies are also considered under profit sharing contracts. For example, Huang et al. [41] evaluated price markdown policies and emphasized their attractiveness for retailers under a revenue sharing contract. Ai et al. [42] created a model featuring two manufacturers and two exclusive retailers to investigate the impact of price risk under a profit-sharing framework. Furthermore, the effects of policy changes, regulations, and unforeseen events on contract execution have been explored [43]. Clearly, revenue-sharing contracts, commonly used in decentralized decision-making frameworks, impact both suppliers’ and retailers’ decisions, mitigating risks from sales uncertainty and boosting supply chain cooperation.

Another commonly practiced contract involves an initial profit allocation, where a pre-determined profit allocation ratio is set between suppliers and retailers under a centralized or cooperative decision-making framework to reduce internal competition and ensure a stable income for all supply chain members, creating a united front against market uncertainty, a practice particularly common in supply chains dominated by large brands [44–46]. Researchers suggest that such contracts have a significant effect on ordering strategies. For example, Xiao et al. [47] considered the supply chain coordination problem under initial profit allocation to enable supply chain members to pursue maximum profits. Gu and Yu [48] established a profit distribution model of integrated agricultural product supply chains using an excess profit distribution coefficient to depict the profit allocation between the retailer and the supplier. Zhou [49] considered a price compensation mechanism under an initial profit distribution ratio and showed that when the retail price is fixed, suppliers and retailers can easily estimate their respective profits, determine their optimal strategies, and demonstrate that the adopted profit distribution can effectively coordinate the supply chain. Although profit allocation practices are widely used, currently, there is no research on the specific effects of market uncertainty, especially price fluctuations combining profit allocation, on ordering strategies.

To sum up, this paper investigates ordering problems under an initial profit allocation within a two-echelon supply chain consisting of a supplier and a retailer. It considers the interactive effects of random price fluctuations, uncertain demand, and profit allocation. Building on existing research, the Copula function is employed to model these fluctuating prices and uncertain demand, and a discrete model is constructed [48–50]. An algorithm is developed to solve an approximate optimal ordering strategy, and its convergence is demonstrated to provide critical support for supply chain decision-making. This study has both academic and practical significance in advancing supply chain decision-making theory in the context of price fluctuation sales to enhance supply chain stability. The main innovations of this study include:

- A supply chain ordering model is developed that considers both initial profit allocation and random price fluctuation, a previously unexplored area, thereby extending the existing ordering theory.
- An algorithm is introduced to approximate the optimal order quantity under dual fluctuations in both price and demand, and proof of convergence is provided.
- The experimental results provide valuable management insights for supply chain members in ordering decisions within a centralized decision-making framework under price fluctuation.

Table 1 presents a detailed comparison with related studies, emphasizing the unique contributions of our research in supply chain ordering.

**Table 1:** Comparison of this research with previous studies on the supply chain ordering problem.

Literature	Strategic retail prices	Price random fluctuations	Profit allocation criterion	Integration of demand uncertainty and price fluctuations
Biswas et al. (2018) [18]	√	-	-	-
Shah et al. (2014) [13]	√	-	-	-
Khouja et al. (2013) [16]	√	-	-	-
Mu et al. (2019) [14]	√	-	-	-
Maiti T and Giri BC (2017) [8]	√	-	-	-
Luo et al. [26]	-	√	-	√
Canyakmaz et al. [27]	-	√	-	√
Gu and Yu (2022) [48]	-	-	√	-
Tapiero and Kogan (2009) [28]	-	√	-	√
Zhou (2020) [49]	-	-	√	-
Yao et al. (2008) [30]	-	-	√	-
Jadidi et al. (2017) [38]	-	-	√	-
Dehghan Bonari et al. (2021) [39]	-	-	√	-
Huang et al. (2022) [41]	√	-	√	-
Zheng et al. (2020) [29]	√	√	-	√
Xiao et al. [47]	-	-	√	-
Xu et al. (2014) [51]	-	√	-	√
Ai et al. (2010) [42]	-	√	√	√
Our research	√	√	√	√

Section 2 delineates the problem and presents the primary hypotheses. Section 3 develops an ordering model for two-level price fluctuations with profit allocation in the supply chain. Section 4 formulates a discrete model and an algorithm for approximating solutions with discrete data. Section 5 presents the results from numerical experiments based on survey data, discussing the impact of various factors on the ordering strategy. Finally, Section 6 summarizes the findings and proposes future research directions.

## 2. Problem description and hypotheses

This paper examines a single-period ordering problem in a supply chain comprising one supplier and one retailer. It focuses on initial profit allocation, a key principle in collaborative supply chains. Profit allocation between the supplier and retailer is addressed from the outset of their transactions to create a community with shared interests, focusing on mutual benefits and maximizing overall supply chain profits. Our goal is to clarify how, under initial profit allocation, the retailer decides the order quantity, and the supplier sets the wholesale price to maximize the supply chain’s total expected profit within a centralized decision-making framework. Besides, due to the widespread presence of random price fluctuations in both online and offline retail environments, the study also examines the impact of random price fluctuations on ordering decisions, particularly within the context of the two-level price fluctuation strategy detailed in the subsequent paragraphs.

Due to frequent fluctuations in online and offline retail prices, this study focuses on a two-level price fluctuation strategy commonly observed in practice. For example, products like fast-moving consumer goods like clothing are typically sold at a regular price level at the beginning of the sales period. However, at the end of the sales season or when inventory levels are too high, retailers implement significant discount strategies, resulting in prices fluctuating around a lower price. This creates two distinct phases: an initial phase where prices fluctuate around the regular price level and a later phase where prices fluctuate around a lower price, i.e., a *two-level price fluctuation* where retail prices fluctuate around two price levels: the regular price and the discounted price.

It is assumed that the retailer sells at a price fluctuating within the range $\left[\eta_{1}^{l},\eta_{1}^{h}\right]$ around the regular level at the beginning of the sales season and then switches to a discounted price range $\left[\eta_{2}^{l},\eta_{2}^{h}\right]$ at the season’s end to boost sales. Given the random nature of retail price fluctuation and uncertain demand, both prices and demands are taken as random variables. To streamline our analysis, the following hypotheses are taken.

1. The retail price at each level is a random variable fluctuating within a continuous range, simplifying the modeling process and accommodating uncertainties in retail prices.
2. The marginal distributions of prices and demands at each level and their joint probability distribution are also continuous.
3. The demands at each price level are independent, a premise adopted in many previous studies [31, 32]. This premise is based on the understanding that some customers prefer buying at regular prices while others wait for discounts. The independence of demand aligns with these varied consumer behaviors, justifying the analysis of sales at different price levels separately.
4. There is no overlap between fluctuation ranges around regular and discount price levels.
5. There is a collaboration between the supplier and the retailer based on a predetermined profit allocation arrangement set in an initial contract.
6. There are no additional losses due to stockouts, and the unit wholesale price is lower than the retail price but higher than the unit production cost.
7. Only a one-time ordering scenario is considered in this study, with replenishment not considered.

Table 2 provides descriptions of the parameters and symbols used.

**Table 2:** Descriptions of parameters and symbols (*t* = 1,2).

Parameters and Symbols	Descriptions
$\eta_{t},\eta_{t}\in [\eta_{t}^{l},\eta_{t}^{h}]$	Fluctuating price at level *t*
*ξ*_*t*_,*ξ*_*t*_≥0	Random demand at level *t*
*h*_*t*_(*η*_*t*_,*ξ*_*t*_)	The joint probability density function of price and demand
*H*_*t*_(*η*_*t*_,*ξ*_*t*_)	The joint probability distribution function of price and demand
*g*_*t*_(*η*_*t*_)	Marginal probability density function of price
*G*_*t*_(*η*_*t*_)	Marginal probability distribution function of price
*f*_*t*_(*ξ*_*t*_)	Marginal probability density function of demand
*F*_*t*_(*ξ*_*t*_)	Marginal probability distribution function of demand
*c*_*t*_(*G*_*t*_(*η*_*t*_),*F*_*t*_(*ξ*_*t*_))	Copula density function
*C*_*t*_(*G*_*t*_(*η*_*t*_),*F*_*t*_(*ξ*_*t*_))	Copula distribution function
*G*_*s*_(*Q*)	Expected profit of supplier
*G*_*re*_(*Q*)	Expected profit of the retailer
$G_{re_{1}}(Q)$	Retailer’s expected profit at the regular level
$G_{re_{2}}(Q)$	Retailer’s expected profit at the discount level
*G*_*sc*_(*Q*)	Total expected profit of the supply chain
*ϕ*	Profit allocation ratio
*w*	Unit wholesale price
*c*_*h*_	Unit disposal cost
*c*_0_	Unit production cost of supplier
*Q*	Order quantity

## 3. Ordering model under two-level price fluctuations with profit allocation

This paper focuses on an ordering problem in a supply chain consisting of a retailer and a supplier, where prices fluctuate at two levels, and the retailer determines the order quantity *Q* by forecasting the fluctuating price and uncertain demand at each price level. To ensure fair profit distribution, an initial profit allocation ratio is established through a contract between the retailer and supplier in a centralized decision-making framework before the sales period. The supplier’s expected profit, *G*_*s*_(*Q*,*w*), is set to be *ϕ* times the retailer’s expected profit *G*_*re*_(*Q*,*w*), which is represented as follows:

$$G_{s}(Q,w)=\phi G_{re}(Q,w)$$ (1)

So *ϕ*, *ϕ*>0 is the profit allocation ratio, indicating the initial allocation of profits between the supplier and the retailer, reflecting their respective bargaining power within the supply chain.*ϕ* = 1 indicates an equal footing, i.e., an even allocation of the total profit in the supply chain. If *ϕ*>1, the supplier acquires a larger share of profit than the retailer. Conversely, *ϕ*<1 signifies the retailer’s advantage.

The supplier’s expected profit function is:

$$G_{s}(Q,w)=(w-c_{0})Q$$ (2)

The retailer’s profit is influenced by uncertain demand and fluctuating retail prices. The demand for the retailer is denoted by *ξ*_1_ at the regular level and *ξ*_2_ at the discount level, and the expected profits for the retailer at the regular and discount levels are represented as $G_{re_{1}}(Q,w)$ and $G_{re_{2}}(Q,w)$ respectively. Then, for the retailer, three potential scenarios arise after comparing the order quantity with the demand from each price level:

Case 1 occurs when the demand at the regular level exceeds the order quantity, i.e., *ξ*_1_≥*Q* all products are sold at the regular price level. No discount strategy is necessary, with no stock at the end of sales. The retailer’s profit in this instance is: (*η*_1_−*w*)*Q*.

Case 2 occurs when the demand at the regular level is less than the order quantity, but the extra is not able to cover the demand at the discount level, i.e., *ξ*_1_<*Q*, and *ξ*_1_≥*Q*−*ξ*_1_. In this case, *ξ*_1_ are sold at the regular level, and all remaining *Q*−*ξ*_1_ are sold at the discount level, again with no stock at the end of the sales. The retailer’s profit, in this case, is expressed as: *η*_1_*ξ*_1_+*η*_2_(*Q*−*ξ*_1_)−*wQ*.

Case 3 occurs when demands at both the regular and discount levels are less than the order quantity, i.e., *ξ*_1_<*Q* and *ξ*_2_<*Q*−*ξ*_1_. In this scenario, *ξ*_1_ and *ξ*_2_ are sold at the regular and discount price levels, respectively. Unsold products incur an additional unit disposal cost of *c*_*h*_ at the end of the sales cycle. The retailer’s profit is then expressed as:

$$\eta_{1}\xi_{1}+\eta_{2}\xi_{2}-c_{h}(Q-\xi_{1}-\xi_{2})-wQ$$

Consequently, the retailer’s expected profit function is:

$$G_{re}(Q,w)=G_{re_{1}}(Q,w)+G_{re_{2}}(Q,w)-wQ,$$

where $G_{re_{1}}(Q,w)$ and $G_{re_{2}}(Q,w)$ are denoted as follows:

$$\left\{ \begin{array}{l} G_{re_{1}}(Q,w)=E[\eta_{1}\times \min\{\xi_{1},Q\}]\\ G_{re_{2}}(Q,w)=E[\eta_{2}\times \min\{Q-\xi_{1},\xi_{2}\}-c_{h}\times {\left(Q-\xi_{1}-\xi_{2}\right)}^{+}] \end{array},\right.$$

where (*a*)^+^ = max{*a*,0}.

Given demand uncertainty and random price fluctuations, price and demand at each level are treated as two-dimensional random variables with their respective joint probability density functions *h*_*t*_(*η*_*t*_,*ξ*_*t*_),*t* = 1,2. Thus, the retailer’s expected profit is expressed as:

$$\begin{array}{l} G_{re}(Q,w)=\int_{\eta_{1}^{l}}^{\eta_{1}^{h}}\int_{Q}^{+\infty }\eta_{1}Qh_{1}(\eta_{1},\xi_{1})d\xi_{1}d\eta_{1}+\int_{\eta_{1}^{l}}^{\eta_{1}^{h}}\int_{0}^{Q}\eta_{1}\xi_{1}h_{1}(\eta_{1},\xi_{1})d\xi_{1}d\eta_{1}\\ \;+\int_{0}^{Q}\int_{\eta_{2}^{l}}^{\eta_{2}^{h}}\int_{Q-\xi_{1}}^{+\infty }\eta_{2}(Q-\xi_{1})h_{2}(\eta_{2},\xi_{2})f_{1}(\xi_{1})d\xi_{2}d\eta_{2}d\xi_{1}\\ \;+\int_{0}^{Q}\int_{\eta_{2}^{l}}^{\eta_{2}^{h}}\int_{0}^{Q-\xi_{1}}\eta_{2}\xi_{2}h_{2}(\eta_{2},\xi_{2})f_{1}(\xi_{1})d\xi_{2}d\eta_{2}d\xi_{1}\\ \;-\int_{0}^{Q}\int_{\eta_{2}^{l}}^{\eta_{2}^{h}}\int_{0}^{Q-\xi_{1}}c_{h}(Q-\xi_{1}-\xi_{2})h_{2}(\eta_{2},\xi_{2})f_{1}(\xi_{1})d\xi_{2}d\eta_{2}d\xi_{1}\\ \;-wQ \end{array}$$ (3)

To maximize the total profit of the supply chain and determine order quantity *Q* and wholesale price *w* under initial profit allocation, the following decision model (P1) is constructed:

$$\begin{array}{l} \max\;G_{sc}=G_{s}+G_{re}\\ \;s.t.G_{s}(Q,w)-\phi G_{re}(Q,w)=0\\ \;Q\geq 0,w\in [\underline{w},\bar{w}] \end{array}$$ (P1)

**Fig 1:**
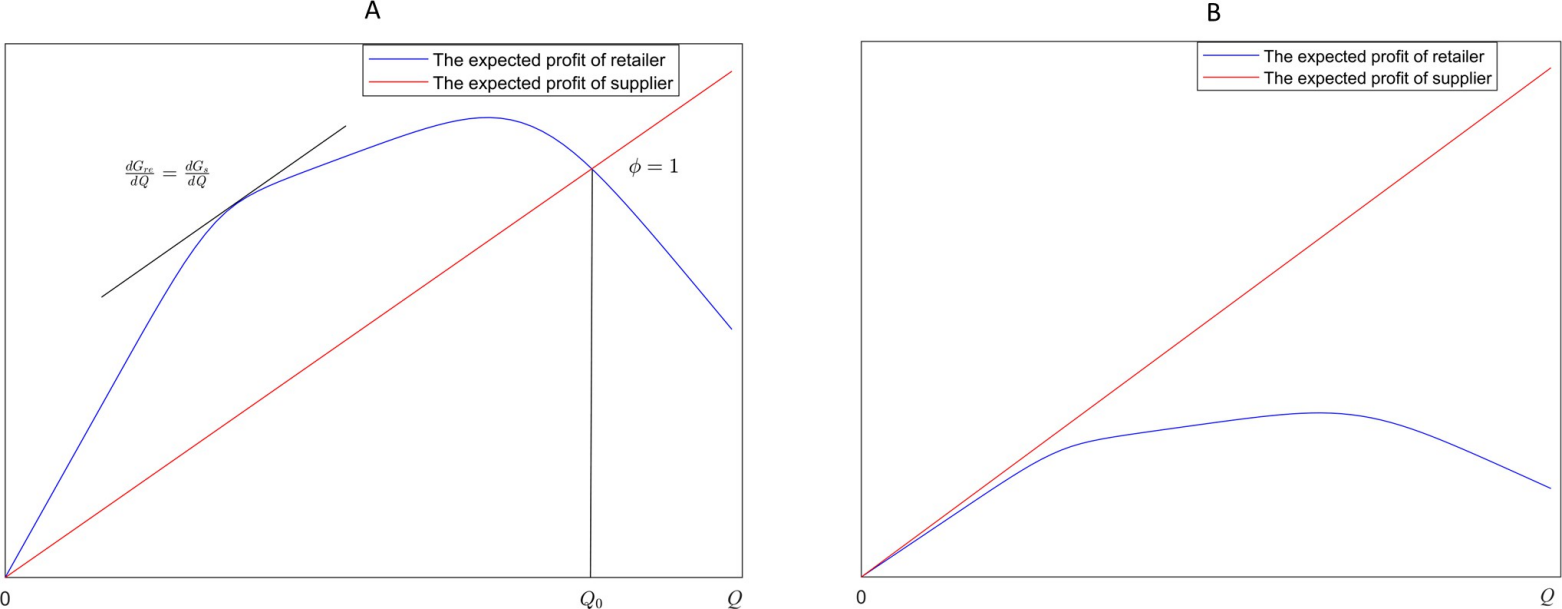
Schematic representation of the relationship between expected profits of the retailer and supplier. (A)Intersection happens at one point. (B)No intersection except at the origin point.

In (P1), $\left[\underline{w},\bar{w}\right]$ is the feasible range for the wholesale price. The expected profits of the supplier and the retailer are defined by (2) and (3). This model suggests that once the initial profit allocation is established, the retailer and the supplier must determine the order quantity and wholesale price using (1). In practice, the supplier and retailer negotiate the allocation, considering their respective advantages. An unfair allocation could hinder practical cooperation. Thus, the potential ranges for *ϕ* should be explored. According to (2) and (3), schematic diagrams illustrating the relationship between the retailer’s and supplier’s expected profits relative to order quantities are given in Fig 1.

Fig 1 depicts two relationships between the supplier’s and retailer’s expected profits at a specific wholesale price. In the first, the expected profit functions intersect at a point other than the origin, as shown in Fig 1A, indicating a balance where both parties have equal profits at a specific order quantity *Q*_0_, i.e., *ϕ* = 1. When *Q*<*Q*_0_, *ϕ*<1, meaning the retailer receives a larger proportion in profit allocation; whereas when *Q*>*Q*_0_, *ϕ*>1, implying the supplier gets a larger share in profit allocation. In the second, the expected profit functions intersect only at the origin, indicating that *ϕ* consistently exceeds 1, attributed to escalated wholesale prices, i.e. the supplier dominates the profit allocation.

Next, conditions that must be met in the first relationship are discussed before Theorem 1 is introduced, laying the groundwork for understanding profit allocation between retailer and supplier.

**Theorem 1:** When joint probability distributions of prices and demands at both levels are continuous, the retailer’s expected profit *G*_*re*_(*Q*,*w*), is a strictly concave function *w*.*r*.*t*. the order quantity *Q* at a given wholesale price *w*.

**Proof of Theorem 1:** Since *h*_*t*_(.), *f*_*t*_(.) are continuous, the variable limit integral function *G*_*re*_(*Q*,*w*) is continuous and differentiable for *Q*>0. Applying the Newton-Leibniz’s Formula, the first-order condition of *G*_*re*_(*Q*,*w*) *w*.*r*.*t*. Q gives:

$$\begin{array}{l} \frac{dG_{re}(Q,w)}{dQ}=\int_{\eta_{1}^{l}}^{\eta_{1}^{h}}\int_{Q}^{+\infty }\eta_{1}h_{1}(\eta_{1},\xi_{1})d\xi_{1}d\eta_{1}+\int_{0}^{Q}\int_{\eta_{2}^{l}}^{\eta_{2}^{h}}\eta_{2}(Q-\xi_{1})h_{2}(\eta_{2},Q-\xi_{1})f_{1}(\xi_{1})d\eta_{2}d\xi_{1}\\ \;+\int_{0}^{Q}\int_{\eta_{2}^{l}}^{\eta_{2}^{h}}\int_{Q-\xi_{1}}^{+\infty }\eta_{2}h_{2}(\eta_{2},\xi_{2})f_{1}(\xi_{1})d\xi_{2}d\eta_{2}d\xi_{1}-\int_{0}^{Q}\int_{\eta_{2}^{l}}^{\eta_{2}^{h}}\eta_{2}(Q-\xi_{1})h_{2}(\eta_{2},Q-\xi_{1})f_{1}(\xi_{1})d\eta_{2}d\xi_{1}\\ \;-\int_{0}^{Q}\int_{\eta_{2}^{l}}^{\eta_{2}^{h}}\int_{0}^{Q-\xi_{1}}c_{h}h_{2}(\eta_{2},\xi_{2})f_{1}(\xi_{1})d\xi_{2}d\eta_{2}d\xi_{1}-w \end{array}$$

After simplifying, we obtain:

$$\begin{array}{l} \frac{dG_{re}(Q,w)}{dQ}=\int_{\eta_{1}^{l}}^{\eta_{1}^{h}}\int_{Q}^{+\infty }\eta_{1}h_{1}(\eta_{1},\xi_{1})d\xi_{1}d\eta_{1}+\int_{0}^{Q}\int_{\eta_{2}^{l}}^{\eta_{2}^{h}}\int_{Q-\xi_{1}}^{+\infty }\eta_{2}h_{2}(\eta_{2},\xi_{2})f_{1}(\xi_{1})d\xi_{2}d\eta_{2}d\xi_{1}\\ \;-\int_{0}^{Q}\int_{\eta_{2}^{l}}^{\eta_{2}^{h}}\int_{0}^{Q-\xi_{1}}c_{h}h_{2}(\eta_{2},\xi_{2})f_{1}(\xi_{1})d\xi_{2}d\eta_{2}d\xi_{1}-w \end{array}$$

The second derivative of the retailer’s expected profit *G*_*re*_(*Q*,*w*) *w*.*r*.*t*. *Q* is:

$$\begin{array}{l} \frac{d^{2}G_{re}(Q,w)}{dQ^{2}}=-\int_{\eta_{1}^{h}}^{\eta_{1}^{h}}\eta_{1}h_{1}(\eta_{1},Q)d\eta_{1}-\int_{0}^{Q}\int_{\eta_{2}^{l}}^{\eta_{2}^{h}}\eta_{2}h_{2}(\eta_{2},Q-\xi_{1})f_{1}(\xi_{1})d\eta_{2}d\xi_{1}\\ \;-\int_{0}^{Q}\int_{\eta_{2}^{l}}^{\eta_{2}^{h}}c_{h}h_{2}(\eta_{2},Q-\xi_{1})f_{1}(\xi_{1})d\eta_{2}d\xi_{1} \end{array}$$

Since *c*_*h*_>0, $\frac{d^{2}G_{re}(Q,w)}{dQ^{2}}<0$. It is observed that the retailer’s expected profit *G*_*re*_(*Q*,*w*) is a strictly concave function *w*.*r*.*t*. *Q* for a given wholesale price *w*.

To provide a foundational understanding of collaborative decision-making within the supply chain, Theorem 2 is introduced, which outlines conditions for both the retailer and supplier to gain potential profit.

**Theorem 2:** The retailer and supplier achieve potential profits if and only if the following equation is satisfied:

$$w<\frac{E(\eta_{1})+c_{0}}{2}$$ (4)

**Proof of Theorem 2:** Building on the proof of Theorem 1, it becomes apparent that when the joint probability distributions of prices and demands are continuous, *G*_*re*_ is a strictly concave function *w*.*r*.*t*. *Q* at a given wholesale price *w*. The first derivative of *G*_*re*_ *w*.*r*.*t*. *Q* is monotonically decreasing, while the first derivative of *G*_*s*_ *w*.*r*.*t*. *Q* is the positive constant *w*−*c*_0_. When *Q* = 0, neither the retailer nor the supplier gets any profit, and their profit functions intersect at the origin. Only when $\frac{dG_{re}(Q,w)}{dQ}{|}_{Q=0}>\frac{dG_{s}(Q,w)}{dQ}{|}_{Q=0}$ the profit functions exhibit another intersection point. Given the conditions $\frac{dG_{re}(Q,w)}{dQ}{|}_{Q=0}=E(\eta_{1})-w$ and $\frac{dG_{s}(Q,w)}{dQ}{|}_{Q=0}=w-c_{0}$, the retailer and the supplier may get their profit proportion if and only if *E*(*η*_1_)−*w*>*w*−*c*_0_.

To find the order quantity and corresponding wholesale price that maximize the profit in (P1), the wholesale price should satisfy condition (4), and the order quantity can be solved through the objective function.

Due to price fluctuation and demand uncertainty, obtaining an analytical solution for the optimal order quantity poses a challenge. Therefore, the following section introduces a discretized model and an algorithm to solve the approximate optimal order quantity.

## 4. Discrete model and solution algorithm for approximate optimal order quantity

Knowing the joint probability distribution of fluctuating prices and uncertain demand is necessary to determine the optimal order quantity. Xu and Li [51] demonstrated that once the marginal distributions of two random variables are known, the Copula function can link them and form a joint distribution. Since the marginal distributions of prices and demand can be easily derived from historical data, the Copula function is applied to capture the correlation between fluctuating prices and uncertain demand. It allows us to model their joint probability distribution and solve the optimal order quantity. Assume the continuous joint probability distribution function is *P*(*x*_1_,*x*_2_) *w*.*r*.*t*. two random variables *x*_1_,*x*_2_, with continuous marginal probability distributions *P*_1_(.) and *P*_2_(.). According to Sklar’s Theorem [50], a unique Copula function exists, denoted as *C*(.,.), satisfying:

$$P(x_{1},x_{2})=C(P_{1}(x_{1}),P_{2}(x_{2}))$$

Thus, given the assumed continuity of marginal distribution functions *F*_*t*_(.) and *G*_*t*_(.), the joint probability distribution functions *H*_*t*_(*η*_*t*_,*ξ*_*t*_) *w*.*r*.*t*. *ξ*_*t*_ and *η*_*t*_ can be uniquely determined (details on Copula function selection and testing are provided in the algorithm and numerical experiments section). Let the Copula function for price and demand be denoted as *C*_*t*_(*G*_*t*_(*η*_*t*_),*F*_*t*_,(*ξ*_*t*_)), with its corresponding Copula density function as *c*_*t*_(*G*_*t*_(*η*_*t*_),*F*_*t*_,(*ξ*_*t*_)). When the Copula function is differentiable, its density function is derived as follows:

$$c(u_{1},u_{2})=\frac{\partial^{2}C(u_{1},u_{2})}{\partial u_{1}\partial u_{2}},$$

where, *u*_1_ = *G*_*t*_(*η*_*t*_),*u*_2_ = *F*_*t*_(*ξ*_*t*_). Subsequently, the joint probability density function is:

$$h_{t}(\eta_{t},\xi_{t})=c_{t}(G_{t}(\eta_{t}),F_{t}(\xi_{t}))g_{t}(\eta_{t})f_{t}(\xi_{t}),t=1,2$$

Consequently, the decision model (P2) is formulated based on the Copula function:

$$\begin{array}{l} \max\;G_{sc\_Copula}\\ \;s.t.G_{s\_Copula}(Q,w)-\phi G_{re\_Copula}(Q,w)=0\\ \;Q\geq 0\\ \;w\in W \end{array}$$ (P2)

In (P2), *G*_*re_Copula*_ represents the retailer’s expected profit under the Copula measure:

$$\begin{array}{l} G_{re\_Copula}=\int_{\eta_{1}^{l}}^{\eta_{1}^{h}}\int_{Q}^{+\infty }\eta_{1}Qc_{1}(G_{1}(\eta_{1}),F_{1}(Q))g_{1}(\eta_{1})f_{1}(Q)d\xi_{1}d\eta_{1}\\ \;+\int_{\eta_{1}^{l}}^{\eta_{1}^{h}}\int_{0}^{Q}\eta_{1}\xi_{1}c_{1}(G_{1}(\eta_{1}),F_{1}(Q))g_{1}(\eta_{1})f_{1}(Q)d\xi_{1}d\eta_{1}\\ \;+\int_{0}^{Q}\int_{\eta_{2}^{l}}^{\eta_{2}^{h}}\int_{Q-\xi_{1}}^{+\infty }\eta_{2}(Q-\xi_{1})c_{2}(G_{2}(\eta_{2}),F_{2}(Q))g_{2}(\eta_{2})f_{2}(Q)f_{1}(\xi_{1})d\xi_{2}d\eta_{2}d\xi_{1}\\ \;+\int_{0}^{Q}\int_{\eta_{2}^{l}}^{\eta_{2}^{h}}\int_{0}^{Q-\xi_{1}}\eta_{2}\xi_{2}c_{2}(G_{2}(\eta_{2}),F_{2}(\xi_{2}))g_{2}(\eta_{2})f_{2}(\xi_{2})f_{1}(\xi_{1})d\xi_{2}d\eta_{2}d\xi_{1}\\ \;-\int_{0}^{Q}\int_{\eta_{2}^{l}}^{\eta_{2}^{h}}\int_{0}^{Q-\xi_{1}}c_{h}(Q-\xi_{1}-\xi_{2})c_{2}(G_{2}(\eta_{2}),F_{2}(\xi_{2}))g_{2}(\eta_{2})f_{2}(\xi_{2})f_{1}(\xi_{1})d\xi_{2}d\eta_{2}d\xi_{1}\\ \;-wQ \end{array}$$

In practice, prices and demand fluctuate among finite, discrete points, and suppliers offer a limited range of wholesale prices. Therefore, the continuous decision model (P2) is transformed into the discrete data model (P3). Discrete values for prices and demand at each level are defined as follows:

$$(\eta_{1i_{1}},\xi_{1j_{1}}),i_{1}=1,2,\ldots ,r_{1},j_{1}=1,2,\ldots ,m_{1}$$

$$(\eta_{2i_{2}},\xi_{2j_{2}}),i_{2}=1,2,\ldots ,r_{2},j_{2}=1,2,\ldots ,m_{2}$$

The joint probability of price and demand at each level is denoted as $P(\eta =\eta_{1i_{1}},\xi =\xi_{1j_{1}})=P_{1i_{1}j_{1}}$, $P(\eta =\eta_{2i_{2}},\xi =\xi_{2j_{2}})=P_{2i_{2}j_{2}}$, with $P_{1j_{1}}$ representing the marginal distribution of demand at the regular level. $\bar{j_{1}}$ is defined as $\bar{j_{1}}\in \{j_{1}|j_{1}=1,2,\ldots ,m_{1}\}$ satisfying $\xi_{1\bar{j_{1}}}\leq Q$ and $\xi_{1(\bar{j_{1}}+1)}>Q$, where $\xi_{1j_{1}}$ ranges from the smallest to the largest value. $\bar{j_{2}}$ is defined as $\bar{j_{2}}\in \{j_{2},j_{2}=1,2,\ldots ,m_{2}\}$ satisfying $\xi_{2\bar{j_{2}}}\leq Q-\xi_{1j_{1}}$ and $\xi_{2(\bar{j_{2}}+1)}>Q-\xi_{1j_{1}}$ at the discount level, where $\xi_{2j_{2}}$ ranges from the smallest to the largest value, and $\xi_{2\bar{j_{2}}}$ is the threshold where the unsold volume after regular price sales meets the demand at the discount level. For discrete data, the profit functions are denoted as ${\bar{G}}_{s}(Q,w)$, ${\bar{G}}_{re}(Q,w)$ and ${\bar{G}}_{sc}(Q,w)$ respectively. This results in model (P3):

$$\begin{array}{l} \max\;{\bar{G}}_{sc}\\ \;s.t.{\bar{G}}_{s}(Q,w)-\phi {\bar{G}}_{re}(Q,w)=0\\ \;Q\geq 0\\ \;w\in W \end{array}$$ (P3)

In (P3) ${\bar{G}}_{s}(Q,w)=G_{s}(Q,w)$, and the expression for ${\bar{G}}_{re}(Q,w)$ is:

$$\begin{array}{l} {\bar{G}}_{re}(Q,w)=\sum_{i_{1}=1}^{r_{1}}\sum_{j_{1}=\bar{j_{1}}}^{m_{1}}\eta_{1i_{1}}QP_{1i_{1}j_{1}}+\sum_{i_{1}=1}^{r_{1}}\sum_{j_{1}=1}^{\bar{j_{1}}}\eta_{1i_{1}}\xi_{1j_{1}}P_{1i_{1}j_{1}}+\sum_{j_{1}=1}^{\bar{j_{1}}}\sum_{i_{2}=1}^{r_{2}}\sum_{j_{1}=\bar{j_{2}}}^{m_{2}}\eta_{2i_{2}}(Q-\xi_{1j_{1}})P_{2i_{2}j_{2}}P_{1j_{1}}-\\ \;+\sum_{j=1}^{\bar{j_{1}}}\sum_{i_{2}=1}^{r_{2}}\sum_{j_{2}=1}^{\bar{j_{2}}}[\eta_{2i_{2}}\xi_{2j_{2}}-c_{h}(Q-\xi_{1j_{1}}-\xi_{2j_{2}})]P_{2i_{2}j_{2}}P_{1j_{1}}-wQ \end{array}$$

Typically, wholesale prices are limited to a discrete range, i.e. *W*. Various price and demand sequences can be simulated using the Copula function, which facilitates finding the optimal order quantity. The following algorithm is presented to determine the approximate optimal order quantity by simulating prices and demand with the Copula function.

Algorithm 1:

Step 1: Based on historical data, determine the marginal distributions of price and demand *G*_*t*_(*η*_*t*_), *F*_*t*_(*ξ*_*t*_), *t* = 1,2 at each level and estimate the relevant parameters.

Step 2: Select a Copula function that adequately describes the correlation between price and demand, initially based on experience, then perform goodness-of-fit tests for validation [52, 53]. Determine the corresponding parameters of the chosen Copula function using historical sales data.

Step 3: Based on the selected Copula function, generate two pairs of random variables (*u*_11_,*u*_12_), (*u*_21_,*u*_22_), with marginal distribution satisfying uniform distribution in [0,1].

Step 4: Obtain values of price and demand through inverse transformation:

$$\eta_{1}=G_{1}^{-1}(u_{11}),\;\xi_{1}=F_{1}^{-1}(u_{12}),\;\eta_{2}=G_{2}^{-1}(u_{21}),\;\xi_{2}=F_{2}^{-1}(u_{22})$$

Repeat Steps 3 and 4 to generate sufficient pairs of price and demand values that satisfy the given marginal distributions and Copula correlation relationship.

Step 5: Calculate demand intervals for each level: for the regular level [min_*Q*_1_,max_*Q*_1_] and for the discount level [min_*Q*_2_,max_*Q*_2_]. Establish the order quantity interval [min_*Q*,max_*Q*], where min_*Q* = min{min_*Q*_1_,min_*Q*_2_} and max_*Q* = max_*Q*_1_+max_*Q*_2_. Based on the generated price-demand pairs, calculate joint probability distributions *P*_*t*_ *t* = 1,2, and analyze marginal distributions of demand $P_{\xi_{t}},t=1,2$. Initialize parameters *c*_*h*_, *c*_0_, *ϕ*, *k* = 1, *N*_*k*_>1, *δ*>0, *ε*>0, and give the wholesale price set *W* = {*w*_*x*_,*x* = 1,2,…*R*}.

Step 6: Check whether the condition $w_{x}<\frac{E(\eta_{1})+c_{0}}{2}$ holds. If it does, take the index set $J_{x}=\{x|w_{x}<\frac{E(\eta_{1})+c_{0}}{2}\}$, *x* = *x*+1, and iterate Step 6.

Step 7: If the index set $J_{x}=\{x|w_{x}<\frac{E(\eta_{1})+c_{0}}{2}\}$ is empty, output: “No intersection point exists at this wholesale price set.” Otherwise, calculate the optimal order quantity ${\bar{Q}}_{x}^{*}$ for the given index set *J*_*x*_.

Step 8: In the range of *Q*, set the step size Δ*Q*_*k*_ = [max_*Q*−min_*Q*]/(*N*_*k*_+1) and select *N*_*k*_ points. Set *Q*_*ik*_ = min_*Q*+*i*Δ*Q*_*k*_ where *i* = 0,1,2,⋯,*N*_*k*_.

Step 9: For each *i*, calculate *Q*_*ik*_ = min_*Q*+*i*Δ*Q*_*k*_where *i* = 1,2,…,*N*_*k*_. According to the constraints of the discrete model (P3), verify if:

$$|{\bar{G}}_{s}(Q_{ik},w_{x})-\phi {\bar{G}}_{re}(Q_{ik},w_{x})|<\delta$$

Include *i* in the index set $I_{k}=\{i||{\bar{G}}_{s}(Q_{ik},w_{x})-\phi {\bar{G}}_{re}(Q_{ik},w_{x})|<\delta ,i=1,2,\ldots ,N_{k}\}$ Step 10: Check if the index set *I*_*k*_ is empty. If yes, output: “No suitable ordering strategy exists under this *w*_*x*_.” Otherwise, identify the set {*Q*_*ik*_} that satisfies the conditions of the index set *I*_*k*_. Calculate $\bar{Q_{ik}^{*}}=\underset{Q_{ik}}{\arg\max}\{{\bar{G}}_{sc}(Q_{ik},w_{x}),k\in I_{k}\}$ and the corresponding supply chain profit ${\bar{G}}_{sc}({\bar{Q}}_{ik}^{*},w_{x})$. Next, check if the convergence condition $|{\bar{G}}_{sc}({\bar{Q}}_{ik}^{*},w_{x})-{\bar{G}}_{sc}({\bar{Q}}_{i(k-1)}^{*},w_{x})|<\varepsilon$ is satisfied. Determine the optimal order quantity $\bar{Q_{x}^{*}}$ that maximizes total profit:

$$\bar{Q_{x}^{*}}=\underset{Q_{ik}}{\arg\max}\{{\bar{G}}_{sc}({\bar{Q}}_{ik}^{*},w_{x}),{\bar{G}}_{sc}({\bar{Q}}_{i(k-1)}^{*},w_{x}),k\in I_{k}\}$$

and the corresponding total supply chain profit $\left\{{\bar{G}}_{sc}(\bar{Q_{x}^{*}},w_{x})\right\}$ under the corresponding *w*_*x*_. If the condition $|{\bar{G}}_{sc}({\bar{Q}}_{ik}^{*},w_{x})-{\bar{G}}_{sc}({\bar{Q}}_{i(k-1)}^{*},w_{x})|<\varepsilon$ is not satisfied, then set *N*_*k*+1_ = *bN*_*k*_, *k* := *k*+1, repeat Steps 9–10.

Step 11: Set *x* = *x*+1, and repeat Steps 8–10 for all *x*∈*J*_*x*_ until the optimal order quantities ${\bar{Q}}_{x}^{*}$ under *w*_*x*_ satisfying the index set *J*_*x*_ are obtained.

Step 12: Gather all order quantities $\left\{\bar{Q_{x}^{*}}\right\}$, and their corresponding total supply chain profits $\left\{{\bar{G}}_{sc}(\bar{Q_{x}^{*}},w_{x})\right\}$, *x*∈*J*_*x*_. Then, obtain the approximate order quantity ${\bar{Q}}^{*}$ that maximizes the supply chain profit under the two-level price fluctuation:

$$({\bar{Q}}^{*},{\bar{w}}^{*})=\arg\;\underset{\bar{Q_{x}^{*}}}{\max}\{{\bar{G}}_{sc}(\bar{Q_{x}^{*}},\;w_{x})\},x\in J_{x}$$

And we obtain the corresponding wholesale price ${\bar{w}}^{*}$.

**Theorem 3:** The supply chain profit sequence ${\bar{G}}_{sc}({\bar{Q}}_{ik}^{*},w_{x})$ obtained by the algorithm converges, ensuring that the algorithm terminates within a finite number of steps.

**Proof of Theorem 3:** According to Step 10, the supply chain profit sequence ${\bar{G}}_{sc}({\bar{Q}}_{ik}^{*},w_{x})$ increases monotonically with *k*. Since *Q*_*ik*_ is bounded, the sequence ${\bar{G}}_{sc}({\bar{Q}}_{ik}^{*},w_{x})$ converges to a finite value. Additionally, since *J*_*x*_ is a finite discrete set, the condition $|{\bar{G}}_{sc}({\bar{Q}}_{ik}^{*},w_{x})-{\bar{G}}_{sc}({\bar{Q}}_{i(k-1)}^{*},w_{x})|<\varepsilon$ is satisfied after a finite number of steps.

In the subsequent section, numerical experiments are conducted to validate the effectiveness and applicability of the proposed discretization model and its approximate algorithm, providing practical strategic recommendations for supply chain decision-makers.

## 5. Numerical experiments and discussion

In this section, the proposed algorithm is applied to give the optimal ordering strategy and the related performance of the supply chain. Based on surveyed sales data, we establish marginal distributions of price and demand and perform goodness-of-fit tests to determine the Copula correlation between them so that our experiment’s price and demand data is generated. First, the approximate optimal order quantity is calculated. Then, the effects of price fluctuations, profit allocations, demand, and Copula correlations on the optimal ordering strategy and the profits of supply chain members are examined, i.e., sensitivity analyses.

### 5.1 Optimal ordering strategy for a seasonal fruit supply chain

A survey of the sales of boxed Dong Kui waxberries was conducted, and they are primarily available from June to July. In this supply chain, the retailer has a long-term partnership with its supplier, and the two agree on a fixed profit allocation ratio at the beginning of each sales season.

Our survey found that the retailer sells waxberries packaged in 500g per box and uses a two-level price strategy: regular and 50% discount, in two sales stages. Statistics indicate that the retail price fluctuates between [82, 99] (CNY per box) at the regular level and [41, 49] (CNY per box) at the discount level. The goodness-of-fit tests indicate a Gaussian Copula correlation between price and demand; thus, the linear correlation coefficient *ρ* is employed to assess the relationship between these variables, where the coefficient value of 0 signifies independence between the variables, 1 indicates a perfect positive relationship, and -1 signifies a perfect negative relationship [54]. Calculations based on historical data determine this coefficient value *ρ* as -0.6.

**Fig 2:**
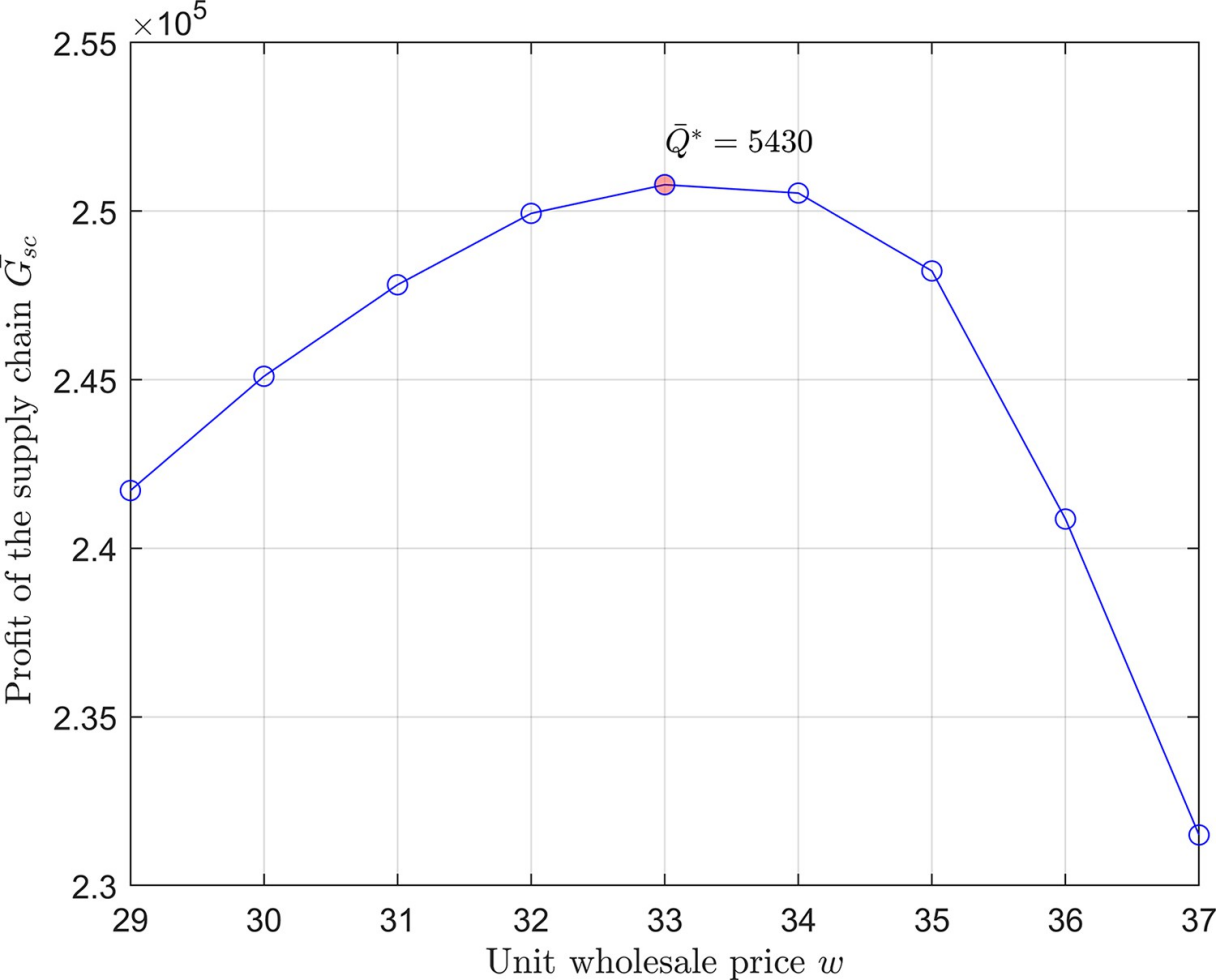
Solution of approximate optimal order quantity.

Using MATLAB and following Algorithm 1 from the previous section, we simulate and generate 5000 pairs of price and demand for each price level, obtaining sufficient data for analysis. It is obtained that the demand at each price level follows a normal distribution with parameters $\mu_{\xi_{1}}=1996$, $\sigma_{\xi_{1}}=197$, $\mu_{\xi_{2}}=3002$, $\sigma_{\xi_{2}}=299.5$, while the price also approximately follows a normal distribution with parameters $\mu_{\eta_{1}}=89.7$, $\sigma_{\eta_{1}}=9.2$, $\mu_{\eta_{2}}=45.2$, $\sigma_{\eta_{2}}=4.5$, respectively. By investigation, set the unit disposal cost *c*_*h*_ = 5 (CNY per box), the unit production cost *c*_0_ = 10 (CNY per box), and the unit wholesale prices *W* = {*w*,*w*∈[10,43]} (CNY per box). Without loss of generality, set *ϕ* = 1 which represents the equal profit allocation between the retailer and the supplier. Fig 2 shows the solution process.

From Fig 2, it is understood that order quantity decreases as wholesale price increases. Concurrently, the overall supply chain profit initially increases, followed by a subsequent decrease. When *w* = 33, the profit of the supply chain reaches its maximum value of 250783, corresponding to an approximate optimal order quantity ${\bar{Q}}^{*}=5430$, the red dot in Fig 2.

### 5.2 Impact of price fluctuations on ordering strategy and profits

This section investigates how price fluctuations impact the optimal order quantity and the profits of supply chain members. In this analysis, the relative standard deviation (RSD), i.e. the percentage of standard deviation from the mean value, is used to describe the degree of price fluctuations.

Our analysis encompasses two scenarios of price fluctuations: 1) The RSD changes occur at the regular level, typically reflecting a scenario where, at the beginning of sales, the retailer is competing for market share and must continuously adjust prices to respond to the market. This leads to fluctuations in the RSD. 2) The RSD changes occur at the discount level, which implies that the retailer is facing inventory pressure or unpredictable customer consumption behavior, resulting in frequent adjustments and intensified fluctuations in discount prices. Without loss of generality, we set the profit ratio *ϕ* = 1. Tables 3 and 4 outline the profits and optimal order quantities for the supply chain across these two price fluctuation changes.

**Table 3:** The impact of price fluctuations at the regular level on profits and the optimal order quantity.

RSD at the regular level (%)	Profit of the retailer	Profit of the supplier	Profit of the supply chain	Optimal order quantity
1	127326	126500	253826	5500
5	126303	125120	251423	5440
10	125893	124890	250783	5430
20	125809	124660	250469	5420
30	124608	123510	248118	5370

**Table 4:** The impact of price fluctuations at the discount level on profits and the optimal order quantity.

RSD at the discount level (%)	Profit of the retailer	Profit of the supplier	Profit of the supply chain	Optimal order quantity
1	126836	125580	252416	5460
5	126726	125810	252536	5470
10	125893	124890	250783	5430
20	119739	118680	238419	5160
30	118711	117760	236471	5120

Tables 3 and 4 collectively demonstrate that an increase in RSD, whether at regular or discount levels, leads to a decrease in the overall profit of the supply chain. Notably, price fluctuations at the discount level have a more pronounced impact on total profit and order strategy than at the regular level. For example, when the RSD increases from 1% to 30%, the retailer’s profit decreases by 6.41% under discount fluctuations while only by 2.13% under regular fluctuations. The decrease in supplier and overall supply chain profits under discount level fluctuations reached 6.23% and 6.33%, respectively, significantly higher than the decrease under regular level fluctuations. These frequent discount price changes are usually caused by inventory pressure or unpredictable consumer behavior, which have a more significant negative impact on the stability of the supply chain.

**Fig 3:**
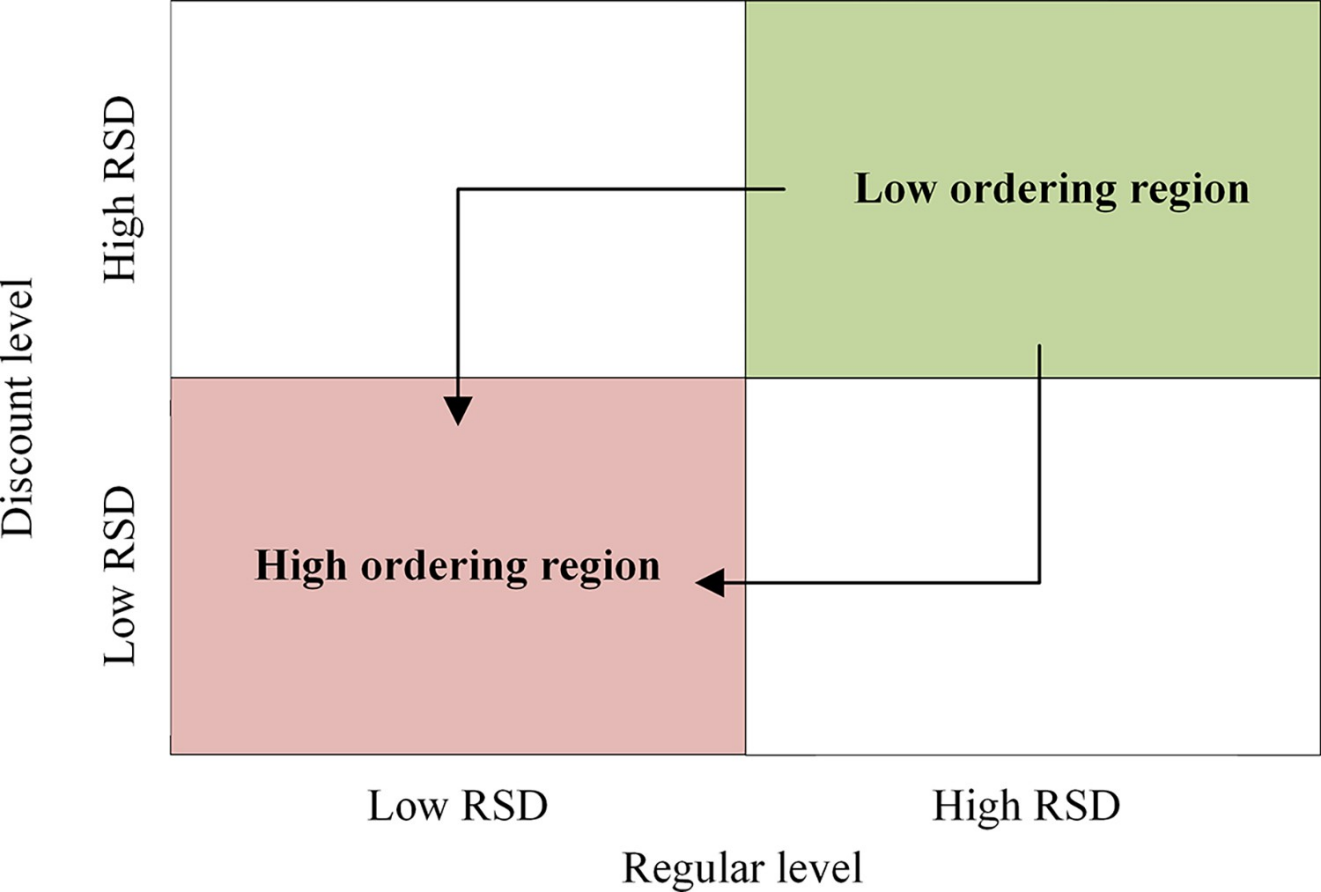
An ordering level typology as price fluctuates at each level.

According to the experiment results above, Fig 3 illustrates ordering strategies facing price fluctuations at each level.

In Fig 3, ordering regions are divided into low-ordering and high-ordering regions. For some products that have just entered the market and are in the early adoption stage with high price uncertainty, supply chain members should choose relatively conservative lower-order quantities to avoid high inventory and market risks. If they quickly expand the market and increase order quantities in high-price fluctuation areas, they will face higher risks but may gain faster and more returns. For some long-term stable products with high market share, due to the long-term existence of the product, the market is saturated and mature, and the original price and discount fluctuations are relatively stable. At this time, a more aggressive ordering strategy can be adopted to increase the order quantity to expand market share and optimize supply chain efficiency. Since price fluctuations at both levels significantly impact the supply chain, managers need to flexibly adjust their strategies to ensure the stability and maximum efficiency of the supply chain.

### 5.3 Effect of profit allocation on decision-making

**Fig 4:**
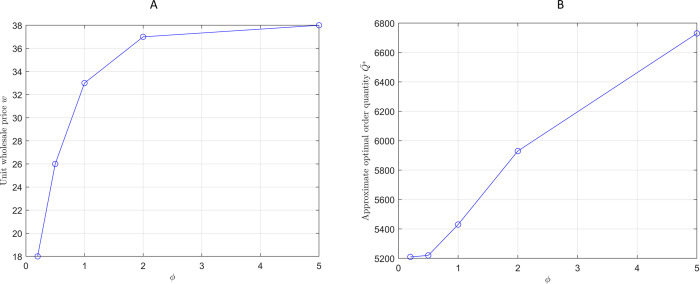
The optimal order quantity ${\bar{Q}}^{*}$ and the wholesale price under different *ϕ*. (A) *w* under the changes of *ϕ*. (B) ${\bar{Q}}^{*}$ under the changes of *ϕ*.

This section explores how profit allocation ratios affect ordering strategies and wholesale prices. Fig 4 analyzes the impact of increasing the profit allocation ratio on optimal order quantities and corresponding wholesale prices.

Fig 4 shows that as the profit allocation ratio increases, the wholesale price rises, but this does not reduce the retailers’ order quantity. Instead, the order quantity increases, reflecting stable cooperation between retailers and suppliers in a centralized decision-making framework. This strategy has been validated in the fast-moving consumer goods industry, where brand owners can optimize the overall supply chain efficiency through close cooperation with suppliers.

### 5.4 Analysis of demand at two price levels and its effect on ordering strategy

When selling under a two-level price strategy, supply chain members face the challenge of uncertain demand due to varying consumer behaviors. Some consumers purchase at the regular price, while others wait for discounts. This section analyzes how demand fluctuations at both levels influence ordering strategies and profits. Assume that the total market capacity remains fixed, but the demand distribution between the regular and discount levels varies. Three demand scenarios are considered, with the ratios of the mean value of demand at the regular to discount levels set at 1:4, 2:3, and 3.5:1.5, respectively. The results are presented in Table 5.

**Table 5:** Optimal order quantities and profits under demand allocation variations.

$\mu_{\xi_{1}}:\mu_{\xi_{2}}$	Optimal order quantity	Profit of the retailer	Profit of the supplier	Profit of the total supply chain
1:4	5400	103242	102600	205842
2:3	5430	125893	124890	250783
3.5:1.5	5600	157959	156800	314759

As shown in Table 5, an increase in the demand ratio at the regular level (from 1:4 to 3.5:1.5) corresponds to an increase in the optimal order quantity. This indicates that higher demand at the regular level necessitates larger order quantities. Concurrently, profits for both the retailer and the supplier increase, suggesting that higher demand at the regular level benefits overall supply chain profit.

### 5.5 Optimizing ordering strategies in different copula-modeled markets

**Fig 5:**
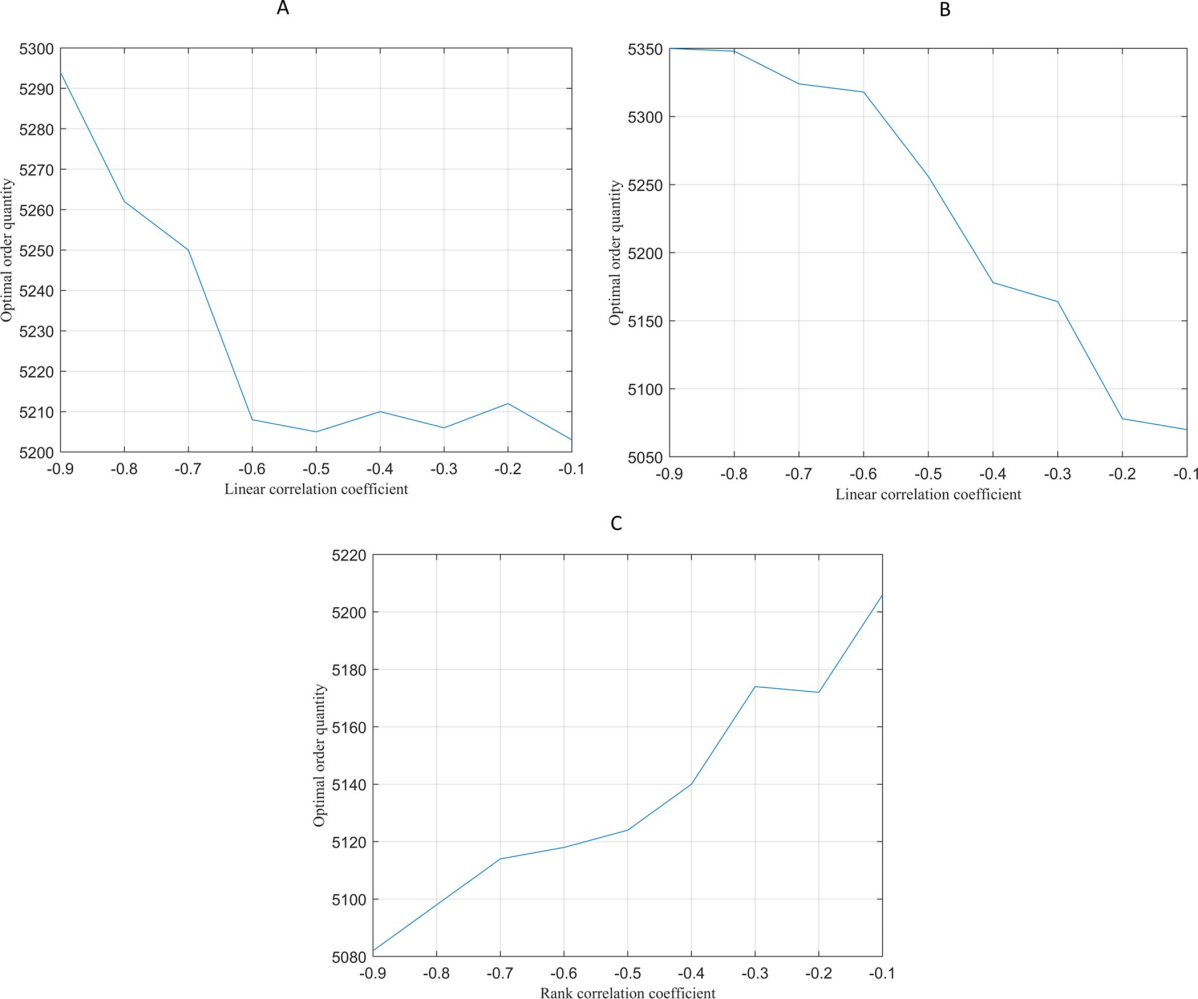
Impact of different Copula functions and correlation strengths on optimal order quantities. (A)The Gaussian Copula function. (B)The t-Copula function. (C)The Frank Copula function.

In previous experiments, the price-demand relationship is characterized by a Gaussian Copula with a correlation coefficient of -0.6. This section examines the impact of different Copula functions (Gaussian, t-Copula, and Frank) and correlation strengths on the optimal order quantity. The Gaussian Copula suits markets with moderate price sensitivity (e.g., daily consumer goods) and shows a linear correlation between price and demand. The t-Copula captures high volatility in markets with price elasticity. The Frank Copula, used for high-end markets (e.g., luxury goods), accounts for a complex, non-linear price-demand relationship where price reductions may not boost demand. MATLAB simulations generated results under these conditions, as shown in Fig 5.

Fig 5 shows that the optimal order quantity decreases rapidly in the Gaussian Copula market as the price-demand correlation increases and then stabilizes. In such markets, maintaining moderate order quantities helps avoid excess inventory. In the t-Copula market, a linear decrease in order quantity is observed, requiring continuous adjustments in volatile markets. Conversely, in the Frank Copula market, the optimal order quantity increases with a higher rank correlation, highlighting that moderate price reductions may not significantly boost demand and can even raise concerns about product quality.

### 5.6 Sensitivity analysis of order quantity relative to disposal and production costs

This section analyzes the optimal ordering strategy’s sensitivity to changes in unit disposal ​ and unit production costs.

**Fig 6:**
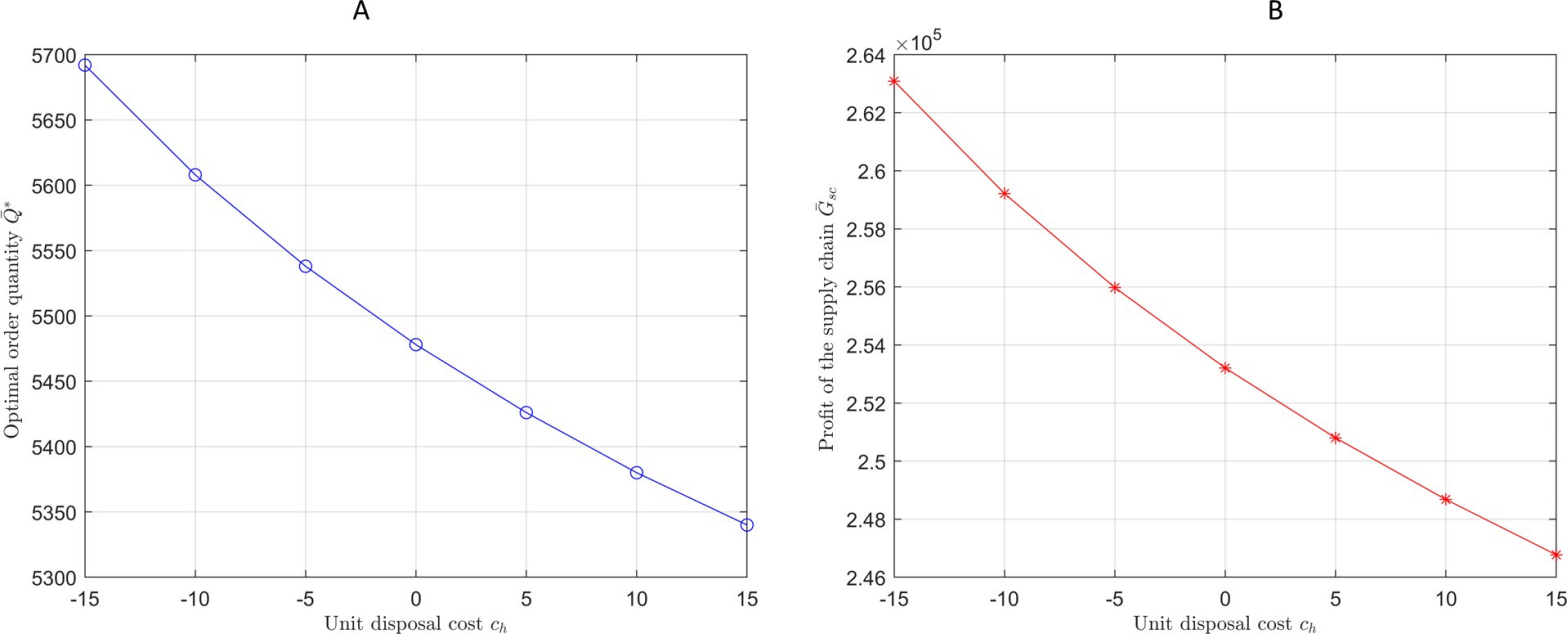
Order quantity and profit of the supply chain under different unit disposal costs. (A)Optimal order quantity under *c*_*h*_ changes. (B)Supply chain profit under *c*_*h*_ changes.

**Fig 7:**
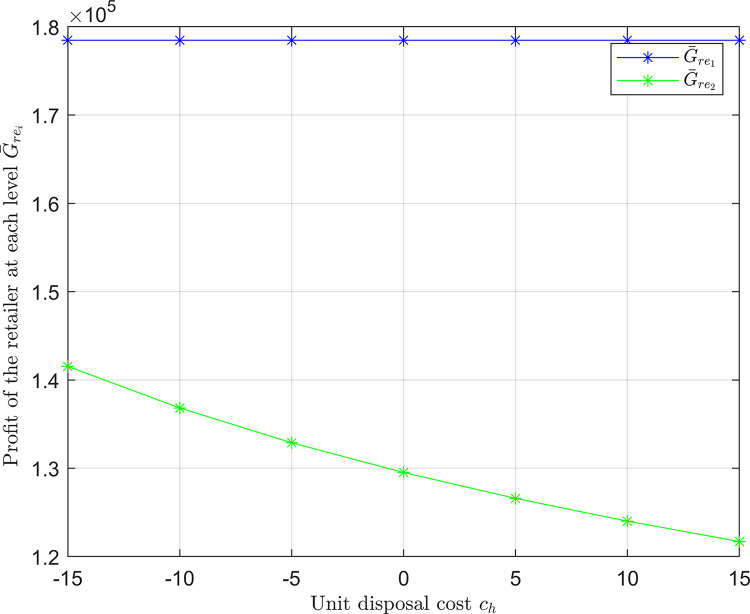
The retailer’s profit at each level is under different unit disposal costs.

First, how changes *c*_*h*_ impact the optimal ordering strategy and the profits of the supply chain members is examined. Research indicates that *c*_*h*_ can also be negative, implying an external revenue or subsidy, referred to as the unit residual value. Set *c*_*h*_ = −15,−10,−5,0,5,10,15. Figs 6 and 7 show the impact of *c*_*h*_ on the optimal ordering strategy and the profits of the supply chain (*ϕ* = 1).

Fig 6A shows rising unit disposal costs decrease optimal order quantities. In contrast, Fig 6B shows a consistent decline in total supply chain profits with increasing disposal costs. Fig 7 highlights that the impact on the retailer’s profit is more significant at the discount level, where rising disposal costs directly reduce profits, while the effect is minimal at the regular price level.

**Fig 8:**
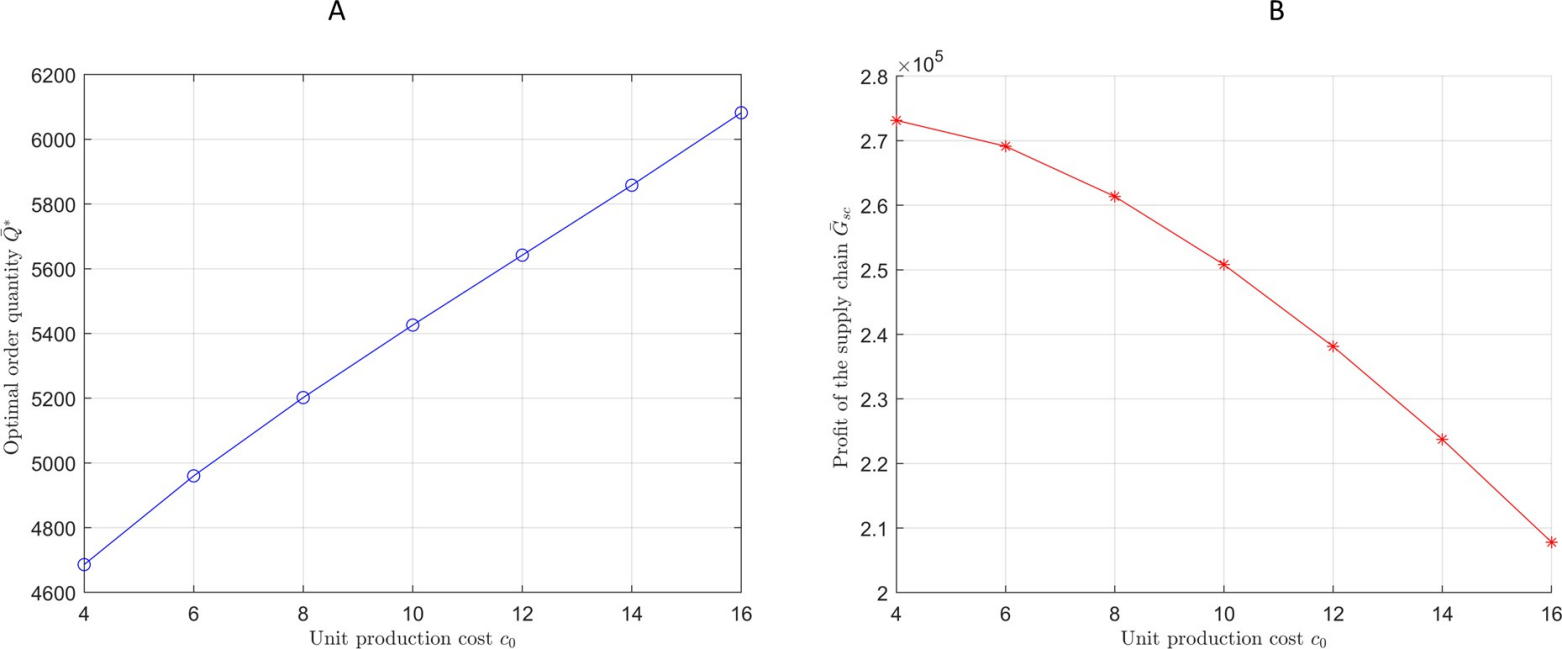
The order quantity and profit of the supply chain under different unit production costs. (A)Optimal order quantity under different *c*_0_. (B)Supply chain profit under different *c*_0_.

Next, we examine how the unit production cost influences the optimal ordering strategy and supply chain profits. The experimental results are presented in Fig 8.

Fig 8A shows that as production costs rise, the optimal order quantity increases to spread out fixed costs and reduce marginal costs, helping to maximize supply chain profit. However, Fig 8B indicates that despite higher order quantities, total profits still decline due to rising production costs. This highlights the need for stricter cost control and innovation to mitigate the negative impact on profit margins.

### 5.7 Managerial insights

Based on the experimental results, several management strategies are proposed to optimize supply chain operations in the face of price fluctuations, demand uncertainty, and profit allocation, which address how supply chain members should adjust their ordering strategies and adapt to fluctuating market conditions.

1) Adjusting ordering strategies in uncertain markets. Price fluctuations, especially at the discount level, significantly impact the supply chain’s profit. Supply chain members should adopt flexible pricing and inventory strategies to manage this. In highly volatile markets, a conservative ordering strategy is recommended to minimize excess inventory and reduce profit losses, while in more stable markets with predictable demand and minimal price fluctuations, an aggressive ordering strategy can help increase market share and improve supply chain efficiency.

2) Formulating ordering strategies under profit allocation contracts. Ordering strategies should account for wholesale pricing and profit allocation dynamics in a profit-allocation-based supply chain. As the supplier’s profit share increases, wholesale prices rise. However, order quantities are maintained or increased with effective coordination and a balanced profit allocation. This approach fosters cooperation and ensures all parties benefit from stable orders and improved supply chain profit.

3) Forecasting demand. Demand shifts between regular and discount levels impact order quantities and profits. Forecasting based on customer preferences and market conditions helps determine optimal order quantities.

4) Managing price fluctuations and demand uncertainty. A conservative ordering strategy can effectively prevent excess inventory and profit losses in markets with high price sensitivity. Conversely, more aggressive ordering strategies can help expand market share in markets less affected by price changes. Supply chain managers should assess market correlations and adjust their ordering strategies accordingly.

5) Optimizing ordering strategies in response to cost sensitivity. Rising production and disposal costs influence order quantities and profits. Supply chain members should balance higher order quantities with the risk of excess inventory and implement strict cost controls.

These managerial insights offer actionable strategies for managing supply chains facing price fluctuations, demand uncertainty, and cost pressures.

## 6. Conclusions and future directions

This study investigates an optimal ordering strategy under initial profit allocation, focusing on two-level price fluctuations. By employing the Copula function to model the correlation between fluctuating prices and uncertain demand, a discrete decision model applicable to real-world scenarios is constructed. An algorithm is proposed to solve the approximate optimal order quantity. Theoretical modeling and numerical experiments yield several key findings:

Price fluctuations, especially at the discount level, significantly affect supply chain profit and ordering strategies. Increased volatility in discount prices can lead to substantial reductions in both the retailer’s and supplier’s profit, highlighting the necessity for adaptive pricing and ordering strategies in uncertain market conditions.

Initial profit allocation has a notable impact on optimal order quantities and the overall profit of the supply chain. Higher profit allocation for suppliers, which increases wholesale prices, does not reduce retailer order quantities under the centralized decision framework.

The demands from regular and discount price levels also play a critical role in determining optimal order quantities. Higher demand at the regular price level typically results in greater overall profit, suggesting that supply chain members should prioritize strategies that maximize sales during the regular price level.

However, this paper still has some limitations. For example, it only considers two-level price fluctuations and focuses solely on profit maximization for simplicity in modeling and solving. As big data and AI technologies evolve, future research could explore ordering strategies in more complex environments, such as multi-stage, multi-channel, and AI-driven price fluctuations [55, 56]. Additionally, much of the current research on ordering strategies emphasize behavioral economics, particularly decision-makers’ utility functions, risk preferences, and loss aversion [17, 28], so future studies could integrate behavioral economics to address ordering decision-making in uncertain environments and use risk preferences as the objective function. Other effective supply chain collaboration criteria could also be explored to enhance decision-making processes [57].

## Supporting information

S1 Dataset The dataset comprises two-level price and sales data generated for numerical experiments. The data were derived from a survey on the sales of boxed Dong Kui waxberries and modeled using the Copula function to simulate realistic price and sales conditions across two levels.
(XLSX)
